# Phenylalanine Regulates Milk Protein Synthesis via LAT1–mTOR Signaling Pathways in Bovine Mammary Epithelial Cells

**DOI:** 10.3390/ijms252313135

**Published:** 2024-12-06

**Authors:** Long Guo, Chen Zheng, Jiao Chen, Ruifang Du, Fei Li

**Affiliations:** 1 State Key Laboratory of Herbage Improvement and Grassland Agro-Ecosystems, College of Pastoral Agriculture Science and Technology, Lanzhou University, Lanzhou 730020, China; guolong@lzu.edu.cn (L.G.); jchen2019@lzu.edu.cn (J.C.); durf21@lzu.edu.cn (R.D.); 2 Animal Nutrition Group, Wageningen University, 6700 AH Wageningen, The Netherlands; chen1.zheng@wur.nl

**Keywords:** phenylalanine, milk protein synthesis, amino acid utilization, bovine mammary epithelial cells, mTOR

## Abstract

Phenylalanine (Phe) is a potentially limiting amino acid for lactating cows. The mechanism by which Phe regulates milk protein synthesis remains unclear. The present study elucidates the mechanisms by which phenylalanine affects milk protein synthesis, amino acid utilization, and related signaling pathways in bovine mammary epithelial cells (BMECs). The BMECs were treated with five concentrations (0, 0.22, 0.44, 0.88, 1.76 mM, and serum free). Rapamycin inhibitors and RNA interference (RNAi) were used to inhibit the phosphorylation of the mammalian target of rapamycin (mTOR) signaling pathway and the expression of relevant amino acid transporters, respectively. The results showed that 4×Phe (0.88 mM) significantly increased (*p* < 0.05) both the mRNA and protein expression of α-casein (*CSN1S1*), β-casein (*CSN2*), and κ-casein (*CSN3*), as well as L-type amino acid transporter-1 (*LAT1*) mRNA expression. Protein expression and modification assays of mTOR-related proteins showed that 4×Phe could increase (*p* < 0.05) the expression of α-casein and eukaryotic initiation factor 4E-binding protein-1 (4EBP1) and tended to increase the expression of ribosomal protein S6 protein kinase (S6K1, *p* = 0.054). The general control nonderepressible 2 (GCN2) signaling pathway factor, eukaryotic initiation factor 2 (eIF2α), was downregulated by 4×Phe treatment (*p* < 0.05). The rapamycin inhibition test showed that Phe regulated casein synthesis via the mTOR signaling pathway. RNAi experiments showed that LAT1 mediated the entry of Phe into cells. Moreover, 4×Phe treatment tended to decrease (0.05 < *p* < 0.10) the consumption of valine, leucine, histidine, tyrosine, cysteine, alanine, asparagine, and serine in the medium. Collectively, phenylalanine enhanced α-casein synthesis by regulating the phosphorylation of 4EBP1 and eIF2α and promoting the formation of the mTOR-centered casein translation initiation complex.

## 1. Introduction

Milk contains essential nutrients, especially high-quality protein, and can fight both viral infection and malnutrition in mothers, infants, children, adolescents, and elderly individuals [1,2,3]. The synthesis of milk protein in the mammary gland requires an adequate supply of energy (adenosine triphosphate) and amino acids (AAs) [4,5]. Lysine (Lys) and methionine (Met) play major limiting roles in dairy production [6], while the next potentially limiting amino acids are phenylalanine (Phe), histidine (His), and arginine (Arg) [7,8]. These AAs can also activate the translational machinery via the signaling pathway and affect the expression of milk protein genes [9,10]. The mammalian target of rapamycin (mTOR) pathway is closely related to milk protein synthesis and provides alternatives for the AA-mediated regulation of milk protein synthesis in bovine mammary epithelial cells (BMECs) [11,12]. The general control nonderepressible 2 (GCN2) signaling pathway is another signaling pathway that senses abundant AA and regulates protein synthesis [13], which senses the absence of one or more amino acids by direct binding to uncharged cognate transport RNAs [14,15]. Eukaryotic initiation factor 2α (eIF2α) is an important downstream factor, and its phosphorylation level directly reflects the activity of the signaling pathway [16]. The above signaling pathways are important for the amino acid regulation of milk protein synthesis [17], but whether Phe affects these pathways has not been reported.

Phe has been shown to affect milk protein production [18]. It has been reported that the mammary gland of dairy cows has a specific requirement for Phe, which increases milk protein production [19]. The supplemented Phe was extracted by the mammary gland in amounts equal to its secretion in milk protein [10,20], which indicates that Phe is almost exclusively utilized to support milk protein production. However, the mechanism by which Phe regulates protein synthesis and AA-mediated cellular signaling in BMECs is still unclear. Based on our previous study, the Phe regulates the synthesis of digestive protein via the mTOR signaling pathway in pancreatic acinar cells [21]. We hypothesized that Phe affects the potential cellular signals to regulate α-casein synthesis. The objective of the present study was to assess the effect of L-Phe on the expression of amino acid transporters as well as on the metabolism of L-Phe in bovine mammary epithelial cells, which produce casein, in an attempt to further elucidate the mechanism by which Phe regulates casein synthesis.

## 2. Results

### 2.1. BMEC Viability and PAH Activity

**Figure 1:**
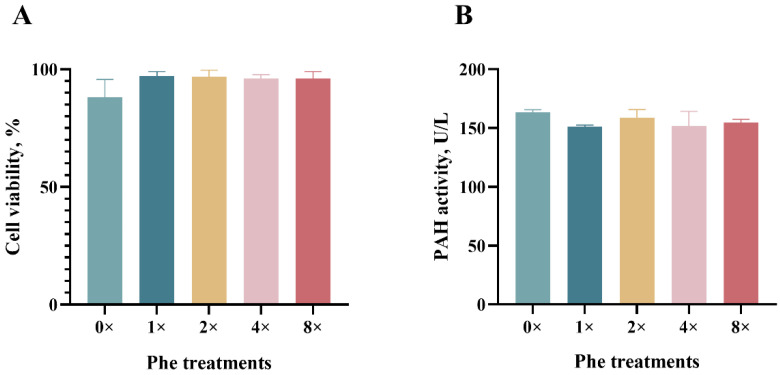
Cell viability and intracellular phenylalanine hydroxylase activity in bovine epithelial cells treated with various concentrations of phenylalanine. (**A**) represents the cell viability. (**B**) represents intracellular phenylalanine hydroxylase (PHA) activity. The error bars represent the SDs (*n* = 3).

There was no significant difference in cell viability among the treatment media (*p* > 0.05, Figure 1A). The PAH activity did not differ among the treatment groups (*p* > 0.05, Figure 1B).

### 2.2. Casein mRNA Expression and Protein Synthesis

**Figure 2:**
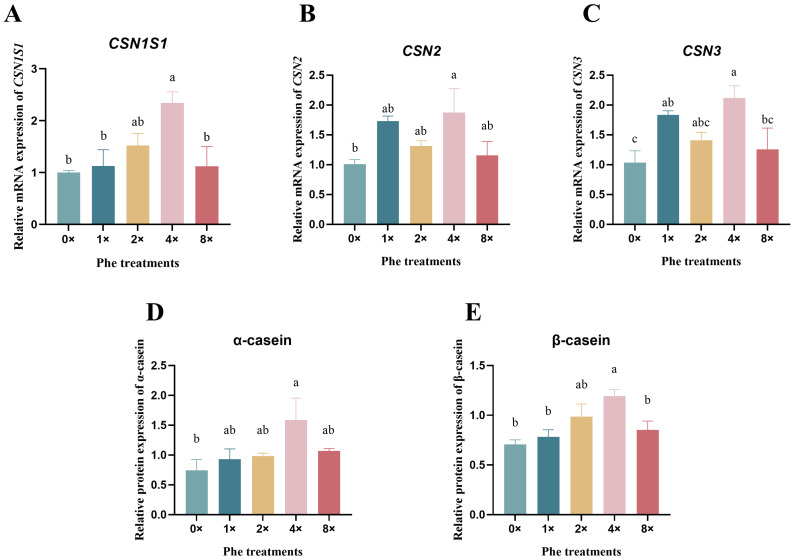
The relative expression of casein mRNA and proteins in bovine mammary epithelial cells treated with various concentrations of phenylalanine. (**A**–**C**) represent the mRNA expression of *CSN1S1*, *CSN2*, and *CSN3*, respectively. (**D**,**E**) represent the protein expression of α-casein and β-casein. The error bars represent the SDs (*n* = 3). Different letters indicate significant differences (*p* < 0.05) (*CSN1S1*, α-casein; *CSN2*, β-casein; *CSN3*, κ-casein).

The mRNA expression levels of *CSN1S1* (Figure 2A), *CSN2* (Figure 2B), and *CSN3* (Figure 2C) in the 4×Phe group were the greatest among all the treatments (*p* < 0.05). Compared with those in the 0×Phe group, both the α-casein (Figure 2D) and β-casein (Figure 2E) protein expression levels in the 4×Phe group increased (*p* < 0.05).

### 2.3. The Expression of Amino Acid Transporters

**Figure 3:**
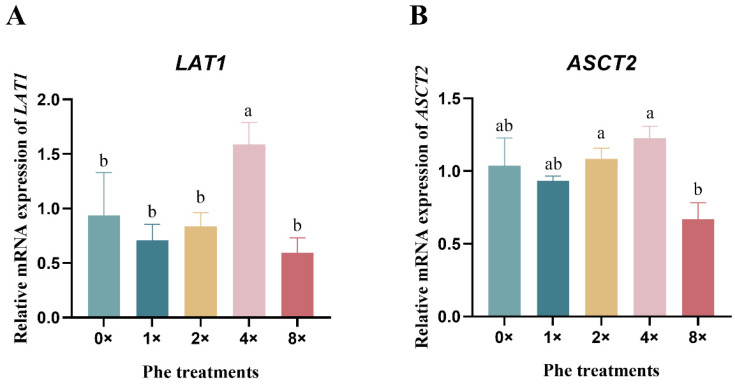
The relative expression of amino acid transporter mRNAs in bovine mammary epithelial cells treated with various concentrations of phenylalanine. (**A**) represents *LAT1*, and (**B**) represents *ASCT2*. The error bars represent the SDs (*n* = 3). Different letters indicate significant differences (*p* < 0.05) (*LAT1*, L-type amino acid transporter 1; *ASCT2*, sodium-dependent neutral amino acid transporter type 2).

The *LAT1* mRNA expression in the 4×Phe group increased (Figure 3A, *p* < 0.05). Compared with the 2×Phe and 4×Phe groups, the 8×Phe group exhibited reduced expression of *ASCT2* mRNA (Figure 3B, *p* < 0.05).

### 2.4. Consumption of Amino Acids by Mammary Epithelial Cells

The results showed that the 4×Phe treatment tended to decrease (0.05< *p* < 0.10, Table 1) the consumption of Val, Leu, His, Tyr, Cys, Ala, Asp, and Ser. There was no significant difference in the other AAs among the treatments (*p* > 0.05).

### 2.5. Mesurement of Phosphorylation Levels of mTOR and GCN2 Signaling Pathway Markers

**Figure 4:**
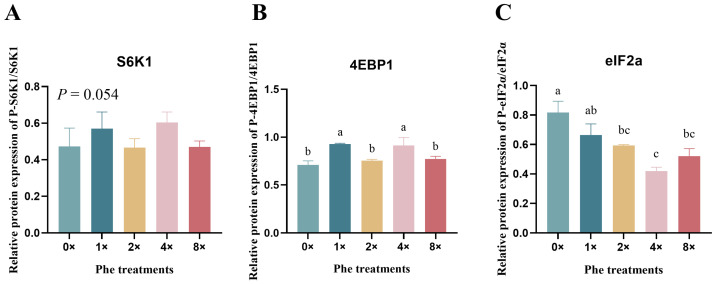
The phosphorylation levels of mTOR and GCN2 signaling pathway factors in bovine mammary epithelial cells treated with various concentrations of phenylalanine. (**A**–**C**) represent the phosphorylation levels of S6K, 4EBP1, and eIF2α, respectively. The error bars represent the SDs (*n* = 3). Different letters indicate significant differences (*p* < 0.05) (S6K1, ribosomal protein S6 protein kinase; 4EBP1, eukaryotic initiation factor 4E-binding protein 1; eIF2α, eukaryotic initiation factor 2α).

We detected the ratio of phosphorylated to total mTOR and GCN2 signaling pathway factors in BMECs treated with various concentrations of Phe (Figure 4). The phosphorylation level of S6K1 tended to increase in the 4×Phe group (Figure 4A, *p* = 0.054). The expression of 4EBP1 was upregulated by 4×Phe and 1×Phe compared with the other treatments (Figure 4B, *p* < 0.05). In contrast, the phosphorylation of eIF2α was significantly lower in the 4×Phe group than in the 0×Phe group (Figure 4C, *p* < 0.05).

### 2.6. Relationships Among Casein Synthesis, Signaling Protein Phosphorylation or Expression, and AA Transporter

The results showed that α-casein tended to be negatively correlated with S6K1 phosphorylation (*p* < 0.05), while β-casein was positively correlated with eIF2 phosphorylation (*p* < 0.05, Table 2).

### 2.7. Rapamycin Inhibitor Expreriment

**Figure 5:**
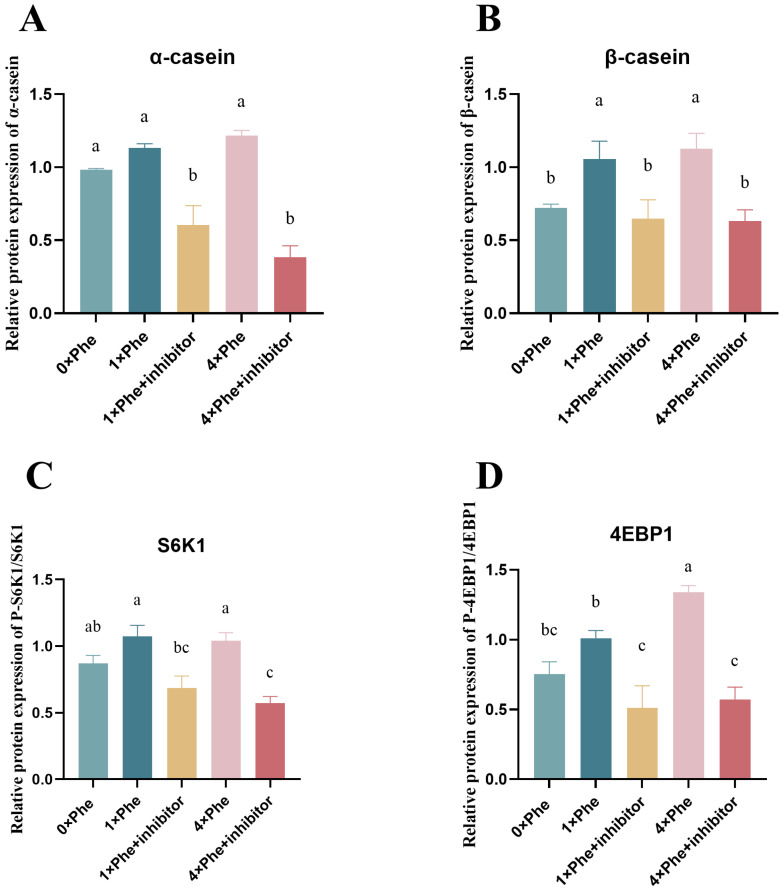
Inhibition of mTOR downregulated S6K1 and 4EBP1 phosphorylation and casein synthesis. (**A**,**B**) represent the protein synthesis of α-casein and β-casein, respectively. (**C**,**D**) show the phosphorylation level of S6K1 and 4EBP1. The error bars represent the SDs (*n* = 3). Different letters indicate significant differences (*p* < 0.05) (S6K1, ribosomal protein S6 protein kinase; 4EBP1, eukaryotic initiation factor 4E-binding protein 1).

The effects of inhibition by mTOR phosphorylation on casein synthesis and phosphorylation of key downstream factors are shown in Figure 5. When treated with the rapamycin inhibitor, the protein expression of α- and β-casein was significantly reduced (Figure 5A,B, *p* < 0.05). The phosphorylation level of S6K1 and 4EBP1 was significantly decreased in the 1×Phe + inhibitor and 4×Phe + inhibitor treatment groups (Figure 5C,D, *p* < 0.05).

### 2.8. Inhibition of LAT1 Decreased Casein Synthesis and mTOR Signaling Pathway Phosphorylation

**Figure 6:**
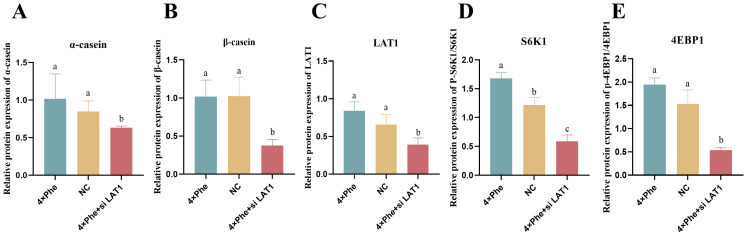
Inhibition of LAT1 downregulated S6K1 and 4EBP1 phosphorylation and casein synthesis. (**A**,**B**) represent the synthesis of α-casein and β-casein, respectively. (**C**) represents the expression of LAT1. (**D**,**E**) show the phosphorylation level of S6K1 and 4EBP1, respectively. The error bars represent the SDs (*n* = 3). Different letters indicate significant differences (*p* < 0.05) (LAT1, L-type amino acid transporter 1; S6K1, ribosomal protein S6 protein kinase; 4EBP1, eukaryotic initiation factor 4E-binding protein 1).

When the cells were treated with siLAT1, the protein abundances of α-casein and β-casein were significantly reduced (Figure 6A,B, *p* < 0.05). The protein expression of LAT1 decreased significantly in the 4×Phe + siLAT1 group (Figure 6C, *p* < 0.05). The phosphorylation levels of S6K1 and 4EBP1 were significantly decreased (Figure 6D,E, *p* < 0.05) after LAT1 inhibition.

## 3. Discussion

### 3.1. Phe Affects Casein Synthesis

The current work elucidates the mechanisms by which phenylalanine affects milk protein synthesis, amino acid utilization, and related signaling pathways in vitro using BMECs. The cell viability was determined in order to measure the adverse effects of the medium on cell growth [22]. In our study, the viability of BMECs was not affected by the addition of Phe, which was consistent with the findings of the previous study [23].

Understanding the regulatory effects of single AA on milk protein synthesis is of great significance in improving the lactating protein and AA requirement models [24]. Lysine and methionine are considered the first and second limiting AAs in corn–soybean meal-based diets [25], while in grass silage-based diets, histidine tends to be the first limiting AA [26]. Therefore, many studies have focused on these three types of AAs on dairy cows [27,28]. Earlier studies showed that branch-chain amino acids (BCAAs) can regulate protein synthesis in dairy cow production [29,30,31]. Moreover, feeding rumen-protected arginine to dairy cows could increase the average daily milk yield [32]. It was also reported that arginine activates the synthesis of milk in sows [33]. The aromatic amino acids Phe and Trp also play important regulatory roles in protein synthesis [34]. The evidence showed that the digestive flow of Phe, on average, was lower than estimated [35]. Our previous study reported that Phe could enhance protein synthesis via the translation pathway in dairy calf pancreatic cells [21]. A recent study showed that when dairy cows got an abomasal infusion of ten EAAs without Phe, milk protein production was lower than that in the Phe-infused group [36], which indicated that Phe may be an important amino acid component that affects milk protein synthesis in dairy cows. The synthesis of milk protein in the mammary gland has specific requirements for Phe [19]; the evidence showed that the absence of Phe in AA infusions negatively affects milk and milk protein yield [8]. These results are consistent with our research, in which the Phe deprivation group negatively affected the gene expression involved in protein synthesis. However, previous research reported that when high milk-yield dairy cows received 7.5 g/cow/day of intestinally absorbable Phe, it was insufficient to support increased milk production because it was primarily used to support an increased body condition score [37]. Our study revealed a significant increase in α-casein synthesis in BMECs after treatment with 0.88 mM Phe in the medium. This result can be explained by differences between individual variations in animals and controlled laboratory environments.

### 3.2. Phe Affects AA Transporter and Cellular Metabolism

The AA uptake by mammary epithelial cells is conducted by different transporters located on the basolateral side of the plasma membrane in the mammary glands [38]. LAT1 (SLC7A5) is a sodium-independent amino acid exchanger that forms a complex with a larger surface glycoprotein, 4F2hc (SLC3A2), to transport several neutral, branched L-type amino acids, such as phenylalanine, tyrosine, and leucine [39]. LAT1 is the most abundant L-type AA transporter in the mammary gland [40]. In addition, LAT1 is involved in the transportation of EAAs, except for arginine, lysine, and threonine [41]. Previous research reported that L-Phe is one of several compounds that possesses a high LAT1 susceptibility, because the phenyl ring of L-phenylalanine provides a high susceptibility to the LAT1 protein [42]. Our results showed that LAT1 expression was positively influenced by the Phe concentration in the medium, which was consistent with the above findings. LAT1 is a key target for mTOR signaling pathway activation [43,44], and we also detected the phosphorylation of mTOR factors, which confirmed that they were positively correlated with LAT1 expression. ASCT2 can transport BCAAs, threonine, and some nonessential AAs to the cell interior [45]. ASCT2 expression showed no obvious change in the present study. This observation is consistent with a former study showing that Phe may not regulate the transport of other AAs into cells [23]. In addition, the high concentration (8×) of phenylalanine in the medium resulted in a decrease in the relative mRNA expression of LAT1 and ASCT2, probably because the addition of Phe inhibited the activity of amino acid transport carriers via a negative feedback mechanism [45].

The total absorption of individual amino acids and the uptake patterns in mammary epithelial cells are affected by the intracellular and extracellular amino acid concentrations and the number or activity of transporters and mammary cells [46,47]. In the present study, the uptake of Phe by mammary epithelial cells increased gradually as the Phe concentration increased, while the consumption of Val, Leu, His, Tyr, Cys, Ala, Asp, and Ser tended to decrease in the 4×Phe treatment group. Combined with the increase in casein synthesis, these results indicated that the AA utilization efficiency was greater in media supplemented with 0.88 mM Phe. Variations in concentrations of extracellular AAs can regulate the transcription, translation, posttranscriptional modifications, and epigenetic regulation of genes and proteins [48]. The results of the lower level of eIF2a phosphorylation indicated that the AA composition of the 4×Phe group was more balanced, which is also the reason for the above results.

PAH is the key rate-limiting enzyme for the conversion of Phe to Tyr [49], which is activated by Phe and then metabolizes excess phenylalanine in cells [50]. The PAH concentration in the mammary epithelial cells in this experiment did not significantly differ among the Phe treatments, especially in the group with a high Phe concentration. We speculated that the additional Phe was mainly involved in the synthesis of milk proteins. Studies of mammalian mammary tissues during lactation have shown that the uptake of Phe is usually balanced with its secretion in milk proteins [51]. When the Phe concentration is elevated, the high substrate concentration induces enzyme activation and promotes Phe catabolism, thereby maintaining the balance of Phe in the blood and providing the body with the required Tyr [49]. A previous publication reported that lactating goat mammary glands convert only 5–9% of the extracted Phe to Tyr, according to a stable isotope perspective [52]. These results suggest that PAH induces little conversion of Phe to Tyr in BMECs.

### 3.3. Phe Regulates Signaling Pathways Related to Casein Synthesis

We further measured the expression of mTORC1 signaling molecules and found that increasing the addition of Phe had a significant effect on the phosphorylation levels of 4EBP1 and S6K1. The mTORC1 signaling pathway controls the main protein synthesis process in mammalian cells [17,53]. When activated by AAs, mTOR in turn catalyzes the phosphorylation of S6K1 and 4EBP1 [54]. The S6K1 protein is a biomarker of mTOR and reflects the phosphorylation of the mTORC1 signaling pathway [55,56]. Phe regulates nucleocytosolic 26S proteasome translocation via mTOR [57], which indicates that Phe is closely related to the mTORC1 signaling pathway. Phe has been reported to regulate the transcription levels of fish target of rapamycin and S6K1 [58,59]. A recent study reported that 1.2 mM Phe in a medium could increase the relative mTOR or S6K1 expression and protein synthesis compared with those in the Phe-deprived group [23], which confirmed our results. A previous study reported that Trp, Phe, and Met had no effect on S6K1 phosphorylation in murine mammary epithelial cells [60]. In the present study, the addition of rapamycin resulted in a significant reduction in the abundance of α-casein and S6k1, indicating that Phe-induced α-casein synthesis via the mTOR pathway was inhibited. However, it has also been shown that the single use of rapamycin inhibitors did not completely inhibit mTORC1 activity and induced the feedback activation of its upstream signaling pathway [61]. Nevertheless, protein synthesis is a highly complex process that involves nutrients, energy status, and hormones [62]. The findings of the current study may help increase our understanding of the roles of Phe in casein synthesis in bovine mammary in vitro.

The GCN2 signaling pathway is sensitive to AA abundance in mammalian cells, which is activated by uncharged tRNA [63], and its biomarker is a downstream factor, eIF2α. GCN2 is activated when an essential amino acid is scarce or unbalanced and phosphorylates the α subunit of eIF2α [64]. The phosphorylation level of eIF2α negatively affects protein synthesis in mammalian cells [65]. In the present study, the phosphorylation level of eIF2α was highest in the 0×Phe group, indicating that the uncharged tRNA of Phe activated the GCN2 signaling pathway. The opposite was true in the 4×Phe group, which indicated that the balance of AA in the medium of the treatment group was more conducive to the synthesis of milk protein. The results of the casein synthesis confirmed these results.

## 4. Materials and Methods

### 4.1. Ethics Statement

The animal experiments in the present study were approved by the Institutional Animal Care and Use Committee at the Institute for Ruminant Research of Lanzhou University (CY-20211101).

### 4.2. Materials

In the present study, penicillin, hydrocortisone, L-Phe, and collagenases were bought from Sigma (Sigma, St. Louis, MO, USA). The fetal bovine serum (FBS) and Dulbecco’s Modified Eagle Medium/Nutrient Mixture F12 (DMEM/F12) were purchased from Invitrogen Company (Invitrogen, Carlsbad, CA, USA). qPCR reagents were purchased from Takara (Takara, Dalian, China). The primary mammary epithelial cells of dairy cows were used in this study.

### 4.3. Cell Culture and Treatment

Three dairy cattle (body weight 724.8 ± 20.88 kg; average lactation parity 3; mean ± SEM) at mid-lactation (lactation days 249 ± 19 d) were used for the BMEC isolation. After the cow was obtained, a 1 cm incision was made with a scalpel in the milking area, and the fascia tissue was stripped, the mammary tissue was exposed, and a small piece (0.5 cm^3^) of mammary tissue was quickly taken with scissors; the tissue was sutured after disinfection. The cells were isolated according to the method of a previous publication using the trypsin digestion technique [66]. Briefly, the mammary gland tissues were minced into 1 mm^3^ cubes and then digested in trypsin (0.25%) at 37 °C for 30 min and subsequently in collagenase I and collagenase II for 4 h at 37 °C.

Bovine mammary epithelial cells (BMECs) were cultured in DMEM/F12 (Cat No. SH30023.01, HyClone, Logan, UT, USA) supplemented with 10% FBS, 100 U/mL penicillin, 0.1 mg/mL streptomycin, and lactating hormones (5 μg/mL insulin, 1 μg/mL prolactin, and 1 μg/mL hydrocortisone) in a humidified 5% carbon dioxide incubator at 37 °C. BMECs purified from passages 5–10 were used for experimental assays. The scanning electron microscope image of mammary epithelial cells of dairy cow were shown in Figure S1.

When the cells were 80% confluent, they were cultured in 6-well plates (1 × 10^6^ cells/well). To observe the effects of Phe on cells, the culture medium was changed to DMEM/F12 medium without FBS. The cells were divided into 5 groups: stripped Phe (0 mM), 1×Phe (0.22 mM, the normal plasma phenylalanine concentration of dairy cows), 2×Phe (0.44 mM), 4×Phe (0.88 mM), and 8×Phe (1.76 mM). After 24 h of incubation, the cells were harvested for further analysis. The experiment was repeated three times on three different days, and six technical replications per group were used on each day, so each experiment had a total of three replicates.

To explore whether mTORC1 signaling participated in the regulation of Phe, the cells were grown in 6-well plates (2 × 10^5^ cells/well) to ~90% confluence and then incubated with 100 nM rapamycin or vehicle [0.02% (vol/vol) dimethyl sulfoxide]. After a 24 h incubation, the cells were collected for further analysis.

### 4.4. Cell Viability Assay

Cell viability was measured using the Cell Counting Kit-8 (CCK8, Cat No. K009-100, Zeta Life, San Francisco, CA, USA) with an Epoch Microplate Spectrophotometer (Synergy^TM^ HTX, BioTek, Winooski, VT, USA), which referred to the previous study [67] and manufacturer’s protocol. Briefly, the procedure was divided into 4 steps: standard curve preparation, cell activity detection, and cell proliferation–toxicity detection and calculation.

### 4.5. Determination of Phenylalanine Hydroxylase Activity

The activity of phenylalanine hydroxylase (PAH) was measured using a commercial kit (ml060412, Shanghai Enzyme-linked Biotechnology Co., Ltd., Shanghai, China) according to the manufacturer’s instructions. An Epoch Microplate Spectrophotometer (Synergy^TM^ HTX, BioTek, Winooski, VT, USA) was used to detect the PAH activity. Briefly, the procedure included sample dilution, mixing, incubation (37 °C, 30 min), washing, enzymatic reaction, rewashing, color development, and determination.

### 4.6. Gene Expression by Real-Time Quantitative PCR (RT-qPCR)

The methods of total RNA extraction and RT-qPCR were performed according to the previous studies of our research group [68]. The total RNA of the cell sample was extracted using TRIzol reagent (Cat No. T9108, Takara, Osaka, Japan). The RNA quality and quantity were measured using a NanoDrop 1000^®^ Spectrophotometer (Thermo Scientific, Waltham, MA, USA). The cDNA was synthesized using Evo M-MLV RT Premix (Cat No. 11706, Accurate Biology, Changsha, China). The RT-qPCR experiment was performed in a 96-well microwell plate using a CFX96 system (Bio-Rad, Herculus, CA, USA). The reactions were performed with a SYBR^®^ Green Premix Pro Taq HS qPCR Kit (Cat No. 11701, Accurate Biology, Changsha, China), with β-actin, GAPDH, and 18S rRNA used as internal controls. The target genes included *ASCT2* (sodium-dependent neutral amino acid transporter type 2), *LAT1* (L-type amino acid transporter 1), *CSN1S1* (α-casein), *CSN2* (β-casein), and *CSN3* (κ-casein), whose primers are shown in Table S1. The running program is as follows: 1 cycle of 95 °C for 30 s plus 40 cycles of amplification at 95 °C for 5 s and 60 °C for 30 s and after the melting curve of an additional 15 s at 95 °C, 1 min at 60 °C and 15 s at 95 °C.

### 4.7. Protein Expression and Modification Analysis

The methods of protein expression were according to our previous study [69]. Total cellular protein was extracted using RIPA lysis buffer (Cat No. R0020, Solabio, Beijing, China) containing phosphatase and protease inhibitors (Roche, Mannheim, Germany). Briefly, the protease and phosphatase inhibitors were mixed with RIPA solution and then added to the cell culture dish after the culture medium was removed and cleaned. The cells were scraped off with a scraper after being shaken on the ice to break the cells and were then transferred to a centrifuge tube for centrifugation. The supernatant was collected as the total protein of the cells. The protein concentration was measured using a Pierce^TM^ BCA assay kit (Thermo Fisher Scientific Inc., Rockford, IL, USA).

Equal amounts of protein extracts (20 μg protein each) were separated on 6–15% SDS-polyacrylamide gels (Bio-Rad, Richmond, CA, USA) and then transferred onto PVDF membranes (Cat No. ISEQ00010, 0.45 μm, Millipore, Bedford, MA, USA) in Tri-glycine buffer containing 20% methanol. The membranes were blocked and immunoblotted with a 1:1000 dilution of primary antibodies against β-tubulin, α-casein, β-casein, p70S6K, P-p70S6K, 4EBP1, P-4EBP1, eIF2α, and P-eIF2α. The target proteins were measured by a goat anti-rabbit IgG or anti-mouse IgG-conjugated secondary antibody (1:3000) with chemiluminescence (ECL) detection reagents (Bio-Rad, Hercules, CA, USA). The antibody information is referred to in Table S2. The Invitrogen iBright Imaging Systems (ThermoFisher Scientific, USA) and ImageJ (https://imagej.net/ij/) were used to detect the protein quantification.

### 4.8. Amino Acid Analysis in Culture Media

The methods of AA analysis were performed according to our previous study [70]. The sixteen AA concentrations in the medium were measured using an AA analyzer (A300 Advanced, MembraPure GmbH, Berlin, Germany). Briefly, the samples were made to react with sulfonyl salicylic acid and then centrifuged, diluted, and filtrated. The AA consumption was calculated as the amount of AA in the media before incubating the cells minus that after incubation.

### 4.9. Statistical Analyses

All data are presented as the means ± standard errors of the means (SEMs) from three independent experiments. The GLM procedure of the Statistical Analysis System 9.2 (SAS Inst. Inc. Cary, NC, USA) was used to perform all of the statistical analyses. The statistical model was as follows: Yi = μ + Ai + Bi, where Yi, μ, Ai, and Bi represent the dependent variable, overall mean, Phe treatment effect, and error term, respectively. Duncan’s test was performed for multiple comparisons of means. A significant difference was defined as *p* ≤ 0.05, and trends were defined as 0.05 < *p* ≤ 0.10. The relative expression of target gene mRNAs was calculated using the 2^−ΔΔCT^ method, and the final result was calculated based on the geometric average of these three internal reference genes. The total protein expression levels were calculated as the ratio of the band intensity of β-actin or β-tubulin.

## 5. Conclusions

Overall, the present study suggested that phenylalanine regulates α-casein synthesis by promoting the LAT1–mTOR pathway in BMECs.

## Figures and Tables

**Table 1:** The consumption of 16 types of amino acids in the medium of bovine mammary epithelial cells.

Item/μg	Initial Mass	0×Phe	1×Phe	2×Phe	4×Phe	8×Phe	SEM ^1^	*p* Value
Lys	273.8	144.0	142.5	143.9	144.3	142.2	0.57	0.126
Phe	-	0.0	69.1	104.8	278.8	155.9	0.41	0.007
Thr	160.4	105.7	104.9	105.6	104.2	104.6	0.34	0.108
Met	51.72	34.1	33.9	34.1	34.4	33.9	0.09	0.105
Val	158.6	104.5	103.9	104.4	102.2	103.7	0.30	0.092
Leu	177.2	116.4	116.2	116.7	114.0	115.9	0.34	0.069
Ile	163.4	108.9	108.8	108.9	109.4	108.5	0.07	0.443
His	94.4	46.2	46.1	46.2	45.5	46.0	0.11	0.068
Arg	442.5	241.0	238.5	240.6	239.7	237.9	0.86	0.118
Gly	53.55	37.0	36.7	37.0	37.0	36.6	0.12	0.138
Tyr	167.4	68.5	68.1	68.5	66.6	67.9	0.26	0.097
Cys	93.9	47.5	46.9	47.29	45.85	46.11	0.23	0.052
Ala	13.5	8.7	8.7	8.7	8.5	8.6	0.04	0.084
Asp	20.0	12.9	12.8	12.9	12.3	12.7	0.09	0.096
Glu	22.1	14.4	14.4	14.5	14.2	14.3	0.06	0.634
Ser	78.8	51.8	51.3	51.7	50.4	51.2	0.19	0.097

^1^ SEM, standard error of the mean.

**Table 2:** Correlations among casein synthesis, signaling protein phosphorylation or expression, and amino acid transporter.

Dependent Variable	Independent Variable	Pearson Correlation	*p* Value
α-casein	β-casein	0.36	0.43
	S6K1	−0.69	0.08
	4EBP1	0.18	0.70
	eIF2a	0.51	0.24
	ASCT2	−0.24	0.61
	LAT1	−0.50	0.25
β-casein	S6K1	−0.45	0.31
	4EBP1	0.16	0.72
	eIF2a	0.75	0.05
	ASCT2	−0.01	0.98
	LAT1	0.09	0.85
S6K1	4EBP1	−0.06	0.90
	eIF2a	−0.69	0.08
	ASCT2	−0.21	0.65
	LAT1	0.20	0.67
4EBP1	eIF2a	−0.12	0.79
	ASCT2	−0.65	0.11
	LAT1	−0.85	0.02
eIF2a	ASCT2	0.30	0.52
	LAT1	0.11	0.81
ASCT2	LAT1	0.68	0.09

## Data Availability

Data is contained within the article and Supplementary Material.

## Supplementary Materials

The following supporting information can be downloaded at: https://www.mdpi.com/article/10.3390/ijms252313135/s1.

## Abbreviations

Phenylalanine, Phe; bovine mammary epithelial cells, BMECs; the mammalian target of rapamycin, mTOR; α-casein, CSN1S1; β-casein, CSN2; κ-casein, CSN3; L-type amino acid transporter-1, LAT1; eukaryotic initiation factor 4E-binding protein-1, 4EBP1; eukaryotic initiation factor 2, eIF2α; amino acids, AAs; lysine, Lys; methionine, Met; isoleucine, Ile; threonine, Thr; histidine, His; arginine, Arg; chemiluminescence, ECL; phenylalanine hydroxylase, PAH; fetal bovine serum, FBS; Dulbecco’s Modified Eagle Medium/Nutrient Mixture F12, DMEM F12.
