# Supplementary material for: Phenylalanine Regulates Milk Protein Synthesis via LAT1–mTOR Signaling Pathways in Bovine Mammary Epithelial Cells
Source: Int J Mol Sci. 2024 Dec 6;25(23):13135. doi: 10.3390/ijms252313135 (PMC11642424; doi:10.3390/ijms252313135)
Supplement: Supplementary file 1 [file ijms-25-13135-s001.zip › ijms-3225518-supplementary.pdf]

Table S1. List of primer sequences for gene expression analysis

Genes	GenBank ID	Primer sequences (5'-3')	Tm	Product length
GAPDH	AJ431207	F:GGCGTGAACCACGAGAAGTATAA<br>R:CCCTCCACGATGCCAAAGT	59.6	98
18S rRNA	NM_QES71278	F:ACCCATTCGAACGTCTGCCCTATT<br>R:TCCTTGGATGTGGTAGCCGTTTCT	59.8	121
β-actin	NM_173979.3	F:AGCAAGCAGGAGTACGATGAGT<br>R:ATCCAACCGACTGCTGTCA	60.1	95
ASCT2	NM_174601.2	F:TGCCGCTGATGATGAAGTGT<br>R:AGTCCACGGCCAAGATCAAG	60.7	86
LAT1	AF174615	F:TACTTCCTTGGGGTCTGGTG<br>R:GTATCTGCGGACATCCACCT	59.5	129
CSN1S1	NM-181029.2	F:TACCTGTCTTGTGGCTGTTGC<br>R:CCTTTTGAATGTGCTTCTGCTC	59.8	112
CSN2	XM-010806178.2	F:AGTGAGGAACAGCAGCAAACAG<br>R:AGCAGAGGCAGAGGAAGGTG	60.4	110
CSN3	NM-174294.2	F:CACCCACACCCACATTTATC<br>R:GACCTGCGTTGTCTTCTTTG	60.1	165
si LAT1	Sense<br>antisense	ACCCTCACTGGTGTTCACG<br>CTCCGGTTTCTGGTAGCG		

ASCT2, sodium-dependent neutral amino acid transporter type 2; *LAT1*, L-type amino acid transporter 1; *CSN1S1*, casein alpha S1; *CSN2*, casein beta; *CSN3*, casein kappa.

Table S2. List of the primary antibodies used in Western blot assay

Antibody	Brand	Cat No.	Production source
β-tubulin	Proteintech	14555-1-AP	USA
β-actin	CWBIO	CW0096M	China
α-casein	Bioss	bs-0813R	China
β-casein	Bioss	bs-10032R	China
p70S6K	Cell Signaling Technology	9202	USA
P-p70S6K	Cell Signaling Technology	9234	USA
4EBP1	Cell Signaling Technology	9452	USA
P-4EBP1	Cell Signaling Technology	9459	USA
eIF2α	Abcam	53245	UK
P-eIF2α	Abcam	33985	UK
goat anti-rabbit IgG	Abcam	Ab6712	UK
Goat anti-mouse IgG	CWBIO	CW0102S	China

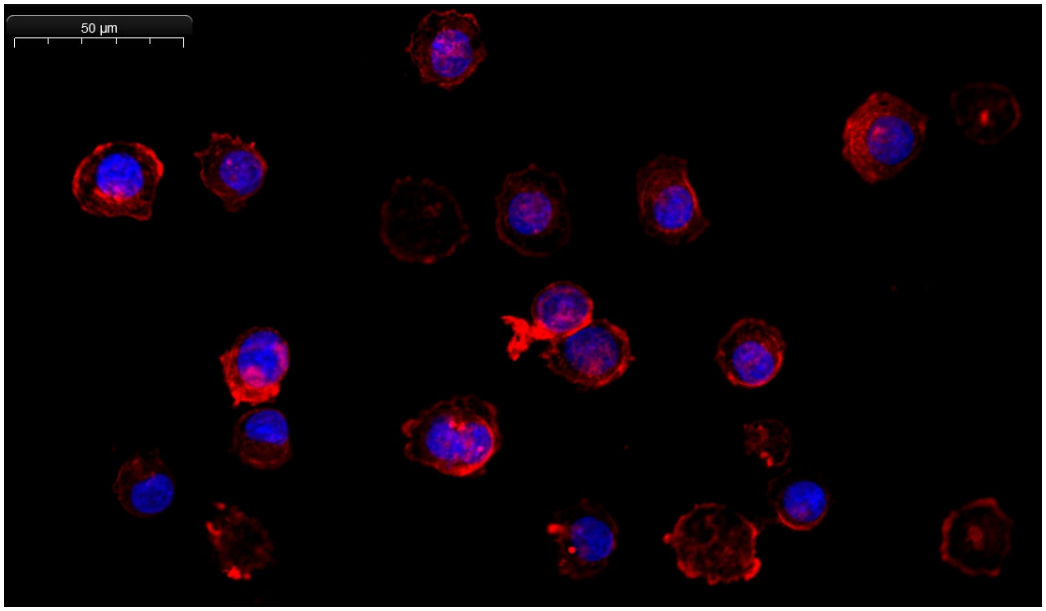

Figure S1. Scanning electron microscope image of mammary epithelial cells of dairy cow.
